# Supplementary material for: Live-cell imaging reveals the trade-off between target search flexibility and efficiency for Cas9 and Cas12a
Source: Nucleic Acids Res. 2024 Apr 22;52(9):5241–56. doi: 10.1093/nar/gkae283 (PMC11109954; doi:10.1093/nar/gkae283)
Supplement: gkae283_Supplemental_File [file gkae283_supplemental_file.pdf]

Supplementary material

Table S1. List of bacterial strains used in this study.

Strains	Characteristics	Source
E. coli DH5a	F^- *endA1 glnV44 thi-1 recA1 relA1 gyrA96 deoR nupG purB20* φ80d*lacZ*ΔM15 Δ(*lacZYA-argF)*U169 *hsd*R17(r_K– m_K+), l^-.	NEB
E. coli PAM-SCANR	*lacIq* $rrnB_{T14}$ $\Delta lacZ_{WJ16}$ hsd_{R514} $\Delta araBAD_{AH33}$ $\Delta rhaBAD_{LD78}$ *rph-1* Δ*cas3*-CRISPR1 ΔP_{lacI}-*lacZ*.	(1)
E. coli PAM-SCANR x p*Spy*dCas9-PAmCherry2.1_Target*,* pTarget	PAM-SCANR strain harbouring plasmids p*Spy*dCas9-PAmCherry2.1_Target and pTarget.	This study
E. coli PAM-SCANR x p*Spy*dCas9-PAmCherry2.1_Scrambled*,* pTarget	PAM-SCANR strain harbouring plasmids p*Spy*dCas9-PAmCherry2.1_Scrambled and pTarget.	This study
E. coli PAM-SCANR x p*Lb*dCas12a-PAmCherry2.1_Target*,* pTarget	PAM-SCANR strain harbouring plasmids p*Lb*dCas12a-PAmCherry2.1_Target and pTarget.	This study
E. coli PAM-SCANR x p*Lb*dCas12a-PAmCherry2.1_Scrambled*,* pTarget	PAM-SCANR strain harbouring plasmids p*Lb*dCas12a-PAmCherry2.1_Scrambled and pTarget.	This study
E. coli PAM-SCANR x p*Sp*RYdCas9-PAmCherry2.1_Target*,* pTarget	PAM-SCANR strain harbouring plasmids p*Sp*RYdCas9-PAmCherry2.1_Target and pTarget.	This study
E. coli PAM-SCANR x p*Sp*RYdCas9-PAmCherry2.1_Scrambled*,* pTarget	PAM-SCANR strain harbouring plasmids p*Sp*RYdCas9-PAmCherry2.1_Scrambled and pTarget.	This study
E. coli PAM-SCANR x pimp*Lb*dCas12a-PAmCherry2.1_Target*,* pTarget	PAM-SCANR strain harbouring plasmids pimp*Lb*dCas12a-PAmCherry2.1_Target and pTarget.	This study
E. coli PAM-SCANR x pimp*Lb*dCas12a-PAmCherry2.1_Scrambled*,* pTarget	PAM-SCANR strain harbouring plasmids pimp*Lb*dCas12a-PAmCherry2.1_Scrambled and pTarget.	This study

Table S2. List of plasmids used in this study.

Plasmid	Relevant characteristics	Source
pBeloBAC11	*repE, ori2, sopABC, cat.*	NEB
pSC101-K102E	*neoR, rep101(K102E).*	(2)
pd*Spy*Cas9-PAmCherry2.1_Target	pBeloBAC11, $P_{LtetO-1}$-BCD-*Spydcas9-PAmCherry2(M10L),* P_{J23119}-*sgRNA(targeting)*.	This study
pd*Spy*Cas9-PAmCherry2.1_Scrambled	pBeloBAC11, $P_{LtetO-1}$-BCD-*Spydcas9-PAmCherry2(M10L),* P_{J23119}-*sgRNA(scrambled)*.	This study
pd*Lb*Cas12a-PAmCherry2.1_Target	pBeloBAC11, $P_{LtetO-1}$-BCD-*Lbdcas12a-PAmCherry2(M10L),* P_{J23119}-*sgRNA(targeting)*.	This study

pd*Lb*Cas12a-PAmCherry2.1_Scrambled	pBeloBAC11, $P_{LtetO-1}$-BCD-*Lbdcas12a--PAmCherry2(M10L)*, P_{J23119}-*sgRNA(scrambled)*.	This study
pSpRYdCas9-PAmCherry2_Target	pBeloBAC11, $P_{LtetO-1}$-BCD-*Spydcas9(A61R, L1111R, D1135L, S1136W, G1218K, E1219Q, A1322R, R1333P, R1335Q, T1337R)-PAmCherry2(M10L)*, P_{J23119}-*sgRNA(targeting)*.	This study
pSpRYdCas9-PAmCherry2_Scrambled	pBeloBAC11, $P_{LtetO-1}$-BCD-*Spydcas9(A61R, L1111R, D1135L, S1136W, G1218K, E1219Q, A1322R, R1333P, R1335Q, T1337R)-PAmCherry2(M10L)*, P_{J23119}-*sgRNA(scrambled)*.	This study
pimp*Lbd*Cas12a-PAmCherry2_Target	pBeloBAC11, $P_{LtetO-1}$-BCD-*Lbdcas12a(D156R, G532R, K538V, Y542R, K595R)-PAmCherry2(M10L)*, P_{J23119}-*sgRNA(targeting)*.	This study
pimp*Lbd*Cas12a-PAmCherry2_Scrambled	pBeloBAC11, $P_{LtetO-1}$-BCD-*Lbdcas12a(D156R, G532R, K538V, Y542R, K595R)-PAmCherry2(M10L)*, P_{J23119}-*sgRNA(scrambled)*.	This study
pTarget	pSC101-K102E, 5 protospacers, 5 30 bp-long distancing sequences.	This study

Table S3. List of oligonucleotides and DNA fragments used to introduce mutations. Nucleotides in bold introduced the listed mutations.

Identifier	Sequence (5'-3')	Used for
BG28281	**AAG**gttatcctcctcgcccttgctc	Introducing M10L mutation in PAmCherry2.1.
BG28278	agggcgaggaggataac**CTT**gccatcatcaaggagttcatgcg	Introducing M10L mutation in PAmCherry2.1.
BG30037	cggaggcttctccaaggaaagtatc**CGC**ccgaaaaggaacagcgacaagctgatcgcacgcaaa<br>aaagattgggaccccaagaaatacggcggattc**CTGTGG**cctacagtcgcttacagtgtactggtt<br>gtggccaaagtggagaaagggaagtctaaaaaactcaaaagcgtcaaggaactgctgggcatcac<br>aatcatggagcgatcaagcttcgaaaaaaccccatcgactttctcgaggcgaaaggatataaaga<br>ggtcaaaaaagacctcatcattaagcttcccaagtactctctctttgagcttgaaaacggccggaaac<br>gaatgctcgctagtgcg**AAACAG**ctgcagaaaggtaacgagctggcactgccctctaaatacgtt<br>aatttcttgtatctggccagccactatgaaaagctcaaagggtctcccgaagataatgagcagaagc<br>agctgttcgtggaacaacacaaacactaccttgatgagatcatcgagcaaataagcgaattctccaa<br>aagagtgatcctcgccgacgctaacctcgataaggtgctttctgcttacaataagcacagggataag<br>cccatcagggagcaggcagaaaacattatccacttgtttactctgacc**CGC**ttgggcgcgcct**CGC**<br>gccttcaagtacttcgacaccaccatagac**CCG**aag**CAA**tac**CGT**tctacaaaggaggtcctgg<br>acg	Introducing L1111R, D1135L, S1136W, G1218K, E1219Q, N1317R, A1322R, R1333P, R1335Q and T1337R mutations of *Sp*RYdCas9.

BG30041	gt**GCG**ttcggccgtctccccg	Introducing A61R mutation of *Sp*RYdCas9.
BG30042	ggggagacggccgaa**CGC**acgcggctcaaaagaacag	Introducing A61R mutation of *Sp*RYdCas9.
BG29090	tatatatgatgcaattcgtaattacgtaacccaaaagccgtacagcaaagataagttcaaactgtatt tccagaacccgcagtttatgCGCggctgggacaaagac**GTT**gagacagac**CGC**cgcgccactat tctgcgttacggcagcaagtactatctcgccatcatggacaaaaaatatgcaaagtgtctgcagaaa atcgataaagacgacgtgaacggaaattacgaaaagattaattataagctgctgccagggcccaac aagatgttaccgCGCgtatttttttccaaaaa	Introducing G532R, K538V, Y542R and K595R mutation of imp*Lb*dCas12a.
BG29087	aacatattttccctgtt**GCG**gaaaaagcccgtgaaggc	Introducing D156R mutation of imp*Lb*dCas12a.
BG29088	**CGC**aacagggaaaatatgttttcagagg	Introducing D156R mutation of imp*Lb*dCas12a.

Supplementary information

Fitting of the histogram derived from Cas nucleases with a scrambled guide

Determination of apparent diffusion coefficient of 3D, 1D and PAM-investigating states

During MC-DDA, initial diffusion coefficients and kinetic rates are provided to initiate a simulation. Several rounds of optimisation are then performed to obtain a fit of the experimental histogram, yielding rates governing the transition between each of the three states present when a Cas nuclease is provided with a scrambled guide. Table S4 lists the apparent diffusion coefficients *D** of each state.

The diffusion coefficient of the nucleases moving in 3 dimensions ($D^*_{3\mathrm{D}}$) was estimated based off their hydrodynamic radius. AlphaFold (3)/ColabFold (4) was used to predict the three-dimensional structures of the fusion proteins *Spy*dCas9-PAmCherry2.1 and *Lb*dCas12a-PAmCherry2.1 (Figure S1). The crystal structures obtained in this way were then used as input for the *in silico* tool HullRad (5). This yielded an hydrodynamic radius of 5.11 nm for *Spy*dCas9-PAmCherry2.1 (corresponding to a diffusion coefficient of ~42 $\mu m^2/s$) and 5 nm for *Lb*dCas12a-PAmCherry2.1 (corresponding to a diffusion coefficient of ~44 $\mu m^2/s$). From here, cytoplasmic retardation was applied to obtain apparent diffusion coefficients D^*_{3D} of 2 $\mu m^2/s$ and 2.2 $\mu m^2/s$ for *Spy*dCas9 and *Lb*dCas12a, respectively.

Regarding 1D diffusion coefficients ($D^*_{1\mathrm{D}}$), we used previous reports on the dynamics of both nucleases obtained through *in vitro* single-molecule approaches. *Spy*dCas9 has been reported to unidirectionally search for a PAM sequence on the DNA by a sliding mechanism of an average length of 20 bp (6, 7). We calculated that the covered distance in this state is ~0.00068 μm (considering an average of 0.000034 μm/bp (8)). This value is lower than our localisation error σ (0.033 μm) and thus a single *Spy*dCas9 is apparently immobile while in its 1D sliding state (D^*_{1D} = 0 $\mu m^2/s$). On the other hand, *Lb*dCas12a has been reported to search for a PAM through a hopping mechanism with an estimated $D^*_{1\mathrm{D}}$ of 1.75 $\mu m^2/s$ (9). Hopping motion consists of intermittent contact with the DNA and as such it can be already potentially described by our model through fast kinetic rates governing transitions between the 3D and the 1D state. Therefore, we also considered *Lb*dCas12a as apparently immobile while in its 1D sliding state (D^*_{1D} = 0 $\mu m^2/s$).

Finally, after encountering a PAM, both *Spy*dCas9 and *Lb*dCas12a molecule stop on the DNA to investigate the protospacer sequence for a match with their gRNA. In this state, the two nucleases are thus immobile and their D^*_{PAM} is 0 $\mu m^2/s$.

Table S4. Diffusion coefficients *D* and apparent diffusion coefficients *D (after cytoplasmic retardation) for *Spy*dCas9, *Lb*dCas12a and their PAM-relaxed variants.**

Nuclease	State	D ($\mu m^2/s$)	Obtained from	D^* ($\mu m^2/s$)
*Spy*dCas9, *Sp*RYdCas9	3D	41.9	Hydrodynamic radius	2
	1D	0	Covered space	0
	PAM	0	Static interaction	0
*Lb*dCas12a, imp*Lb*dCas12a	3D	44.1	Hydrodynamic radius	2.2
	1D	0	Covered space	0
	PAM	0	Static interaction	0

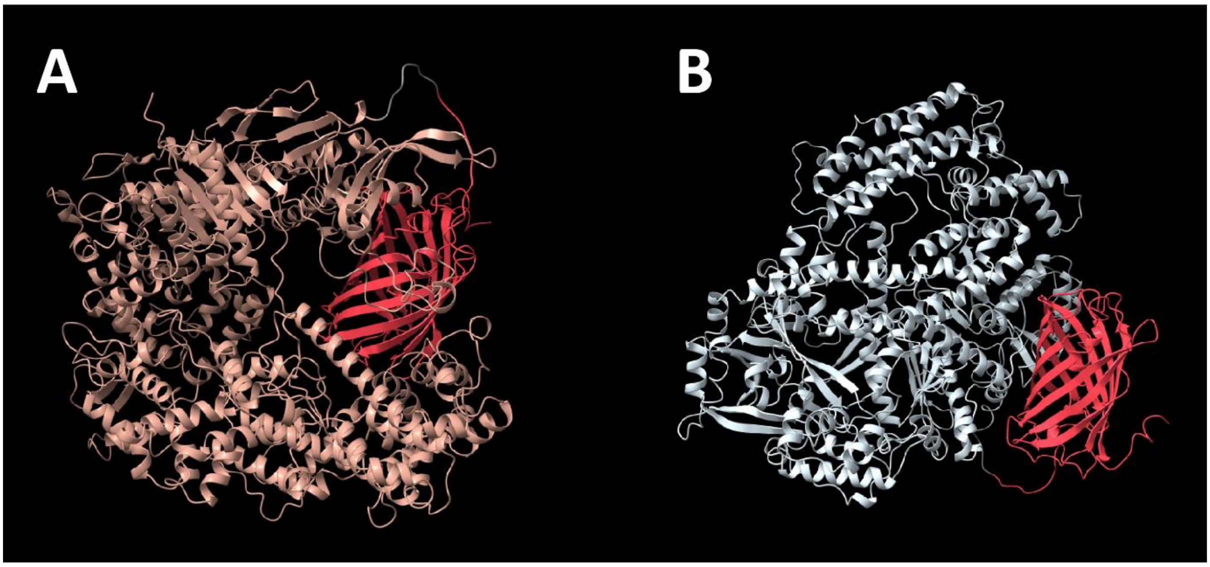

Figure S1. AlphaFold-predicted 3D structures of A) *Spy*dCas9-PAmCherry2.1 and B) *Lb*dCas12a-PAmCherry2.1. *Spy*dCas9 is coloured in light red, *Lb*dCas12a is coloured in light blue, the linker sequences are coloured in grey and the photoactivatable fluorescent protein PAmCherry2.1 is coloured in bright red.

The experimental diffusional histograms (main text Figure 2, Figure 3, Figure 5) show tracks that rarely reach the D^*_{3D} values mentioned above. This observation is explained by the rapid transitioning of molecules between the fast, 3D state and the slow, 1D or PAM-investigating states. Since we assign a single diffusion coefficient per track, corresponding to the average of the diffusion coefficients of each step of the track, only tracks derived from the rare molecules that are in 3D state for the entire duration of the track would yield diffusion coefficients higher than 2 $\mu m^2/s$.

Initialisation of simulated particles

At the beginning of MC-DDA, an initial set of kinetic rates ($k_{3D \to 1D}$, $k_{1D \to 3D}$, $k_{1D \to PAM}$, $k_{PAM \to 1D}$) are provided as an initial guess to interpret the distribution. These rates are used to determine the starting state of each simulated protein as follows:

$$c_{3D} = \frac{k_{PAM\to 1D} \cdot k_{1D\to 3D}}{(k_{1D\to PAM} \cdot k_{3D\to 1D}) + [k_{PAM\to 1D} \cdot (k_{1D\to 3D} + k_{3D\to 1D})]}$$

$$c_{1D} = \frac{k_{PAM\to 1D} \cdot k_{3D\to 1D}}{(k_{1D\to PAM} \cdot k_{3D\to 1D}) + [k_{PAM\to 1D} \cdot (k_{1D\to 3D} + k_{3D\to 1D})]}$$

$$c_{PAM} = \frac{k_{1D\to PAM} \cdot k_{3D\to 1D}}{(k_{1D\to PAM} \cdot k_{3D\to 1D}) + [k_{PAM\to 1D} \cdot (k_{1D\to 3D} + k_{3D\to 1D})]}$$

Where c is the probability of a protein being in the specific state, with $c_{3D} + c_{1D} + c_{PAM} = 1$.

Prediction of target search time

Variables used in the prediction of target search time of Cas nucleases

To estimate the probability that one of the studied Cas nucleases is able to find a single target in a specific cell, our software takes into consideration several variables. The user provides the time available to the nuclease, the number of units to simulate per cell, the GC content of the organism, the specific PAM sequence, the number of times to repeat the simulation, the size and average copy number of the genome, the size and copy number of the pTarget plasmid and the dissociation probability P_{dis}. This latter value is defined as $P_{dis} = \frac{1}{s^2}$, where s is the sliding length of the Cas nuclease in 1D motion. To estimate P_{dis}, we used the previously reported probabilities of *Spy*dCas9 transitioning between adjacent PAMs when the distance between these motifs was increased(6). We fitted this data using exponential decay and obtained $P_{dis} \approx 0.144$ (Figure S2), corresponding to an average s = 3 bp. Due to the lack of experimental characterisation of 1D sliding length for the remainder of the investigated proteins, we considered all Cas nucleases having the same average sliding length s and P_{dis} of *Spy*dCas9.

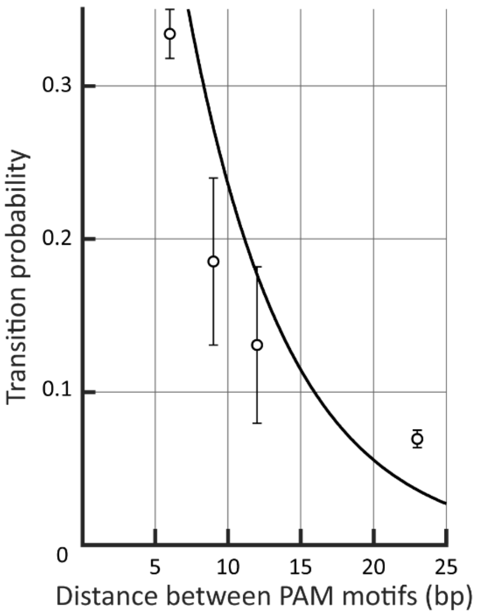

Figure S2. Exponential decay fit of *Spy*dCas9 transition probability between adjacent PAM motifs. The transition probabilities between PAM motifs with varying distances previously described(6) were fitted with an exponential decay to determine the P_{dis} value (black line $y = e^{-0.144x}$).

From the input data, several other nuclease- and host-specific values are calculated and used (the complete set is available in Table S5). Two values are obtained through the GC content of the organism, the size of genome and pTarget plasmid and their average copy numbers: 1) the average number of PAMs; 2) the chance that a random stretch of N nucleotides matches the PAM sequence (P_{PAM}), where N is the PAM sequence length. Now knowing P_{PAM}, one can estimate the average number of PAMs a nuclease encounters when sliding on the DNA (n_{1D_PAM}) for a length *s* (and thus a dissociation probability P_{dis}), through:

$$n_{1D\_PAM} = 1 + 4 \cdot P_{PAM} \cdot \sqrt{\sum e^{\ln(1-P_{dis})}}$$

Here, the sum corresponds to the average number of times the protein will move to the base situated left or right from the base it is currently on. Since it is a diffusional process, the square root is used to find the average sliding length, corresponding to the region where the protein searches for PAM sequences. One factor 2 is added to account for the effect that the protein can slide either way, and another factor 2 because the protein can bind on either one of two strands of the DNA.

Table S5. List of values used for *Spy*dCas9, *Sp*RYdCas9, *Lb*dCas12a and imp*Lb*dCas12a for the simulation of target search. For imp*Lb*dCas12a, recognising PAMs not collapsible in one notation (5'-TNTN, 5'-TACV, 5'-TTCV, 5'-CTCV, 5'-CCCV), the average number of each motif was obtained and the sum was used during the simulation. In the same way, the P_{PAM} value used is a sum of the P_{PAM} value of each individual PAM sequence.

	**Cas nuclease**			
**Variables**	***Spy*dCas9**	***Sp*RYdCas9**	***Lb*dCas12a**	**imp*Lb*dCas12a**
Genome size (bp)	4,654,359	4,654,359	4,654,359	4,654,359
Genome copies	2.3	2.3	2.3	2.3
pTarget size (bp)	3,868	3,868	3,868	3,868
pTarget copies	1	1	1	1
P_{dis}	0.144	0.144	0.144	0.144
PAM sequence (5'-3')	5'-NGG	5'-NNN	5'-TTTV	5'-TNTN, 5'-TACV, 5'-TTCV, 5'-CTCV, 5'-CCCV
Average PAM count	1,365,518	21,417,790	244,347	2,324,340
P_{PAM}	0.0638	1	0.0114	0.1085
$n_{1D_{PAM}}$	1.6201	10.7253	1.111	2.0552

Simulation of target search

Each nuclease unit was individually simulated as mentioned for the MCDDA of diffusional histograms of Cas proteins with scrambled guides. As mentioned before, a t_{Change} times (Materials and methods: Monte-Carlo diffusion distribution analysis of Cas nucleases with a scrambled guide) and initial state of the simulated unit (Supplementary information: Initialisation of simulated particles) were assigned according to the specific rates for transitions between states. When in 1D state, the nuclease had three potential fates. A random number *p* (with 0 < *p* < 1) was generated and used to determine the fate of the unit.

First, a p ≤ $P_{target} = \frac{nr\ target\ sites - nr\ bound\ proteins}{nr\ PAM\ motifs} \cdot n_{1D_{PAM}}$ meant the nuclease found a PAM immediately followed by a matching protospacer, entering an irreversibly bound state. We only simulated the presence of 1 target site, thus ending the simulation for the current cell.

Second, if p ≤ $(c_{PAM} \cdot n_{1D_{PAM}})$ a PAM was encountered, yet not followed by the correct protospacer. A new t_{Change} was then assigned according to the $k_{PAM \rightarrow 1D}$ rate and the simulation continued.

If none of the previous cases was true, the nuclease was considered as reverting to its 3D state after not having found any PAM sequence. Its t_{Change} was thus changed according to the $k_{3D \rightarrow 1D}$ rate and the simulation continued.

Source data

Source Data Table 1. Source data of non-specific interaction times (t_{NSI}), used for the bar plot of Figure 2C.

Nuclease	t_{NSI} (ms)	Uncertainty
*Spy*dCas9	22.0	1.5
*Sp*RYdCas9	32.7	2.6
*Lb*dCas12a	10.2	0.3
imp*Lb*dCas12a	16.6	1.5

Source Data Table 2. Source data of PAM-investigating fractions, used for the bar plot of Figure 2D. The other fractions, totalling to 1, are also reported.

	PAM-investigating		1D		3D	
**Nuclease**	**Prob (%)**	**St. dev.**	**Prob (%)**	**St. dev.**	**Prob (%)**	**St. dev.**
*Spy*dCas9	0.330709	0.03501	0.275591	0.030371	0.393701	0.036079
*Sp*RYdCas9	0.338498	0.031611	0.300177	0.02838	0.361325	0.034974
*Lb*dCas12a	0.351324	0.016209	0.198574	0.009969	0.450102	0.018814
imp*Lb*dCas12a	0.469588	0.030267	0.141941	0.010746	0.38847	0.02197

Source Data Table 3. Source data of average diffusion coefficients (μm²/s), used for the scatter plots of Figure 3B.

	*Spy*dCas9		*Sp*RYdCas9		*Lb*dCas12a		imp*Lb*dCas12a	
**Track length**	**Avg. *D****	**St. dev.**	**Avg. *D****	**St. dev.**	**Avg. *D****	**St. dev.**	**Avg. *D****	**St. dev.**
3	0.57421921	0.006045	0.542011	0.00624	0.784748	0.008135	0.69277	0.007546
4	0.48265905	0.005354	0.4458916	0.005573	0.737751	0.007861	0.6554	0.00823
5	0.42657774	0.004586	0.3827837	0.004809	0.718802	0.007629	0.628498	0.008021
6	0.38228715	0.004047	0.3557168	0.004466	0.736711	0.007028	0.62063	0.007804
7	0.36854012	0.003393	0.3315628	0.004071	0.72226	0.005696	0.600112	0.007397
>8	0.39834597	0.002726	0.333857	0.005361	0.734685	0.004565	0.630357	0.00624

Source Data Table 4. Source data of average probability of 1 unit of each Cas nuclease to find 1 target inside the *E. coli* genome, used for scatter plot of Figure 4A (continuous lines).

	*Spy*dCas9		*Sp*RYdCas9		*Lb*dCas12a		imp*Lb*dCas12a	
**Time (min)**	**Avg. prob.**	**St. dev.**	**Avg. prob.**	**St. dev.**	**Avg. prob.**	**St. dev.**	**Avg. prob.**	**St. dev.**
1	0.33	0.1006	0.2	0.0943	2.065	0.1617	0.44	0.163
5	1.645	0.1536	0.935	0.231	10.68	0.4934	2.195	0.3647
10	3.15	0.3291	1.7	0.2896	19.575	0.5181	4.075	0.5361
30	9.76	0.8171	5.54	0.395	49.93	1.3907	11.94	0.6306
60	18.525	0.8545	10.39	0.6501	74.1	0.5788	21.62	0.68
120	33.015	1.1188	19.71	0.914	93.315	0.7835	39.115	0.886
240	55.15	1.0904	35.315	1.0483	100	0	63.755	0.7286
480	79.99	0.8736	59.04	0.7923	100	0	86.57	0.7436

Source Data Table 5. Source data of average probability of 10 units of each Cas nuclease to find 1 target inside the *E. coli* genome, used for scatter plot of Figure 4A (dashed lines).

	*Spy*dCas9		*Sp*RYdCas9		*Lb*dCas12a		imp*Lb*dCas12a	
Time (min)	Avg. prob.	St. dev.	Avg. prob.	St. dev.	Avg. prob.	St. dev.	Avg. prob.	St. dev.
1	3.41	0.3044	1.69	0.2923	20.735	0.6876	4.155	0.376
5	15.45	0.7785	8.665	0.5391	68.29	1.0096	18.795	1.0114
10	28.675	1.1304	16.825	1.0191	89.49	0.6096	33.66	1.0456
30	63.54	0.667	42.65	1.2383	99.89	0.0394	71.685	0.6684
60	86.995	0.8821	67.02	0.7173	100	0	91.87	0.4015
120	98.175	0.3129	88.74	0.9101	100	0	99.315	0.2274
240	99.965	0.0412	98.95	0.2646	100	0	100	0
480	100	0	99.99	0.0211	100	0	100	0

Source Data Table 6. Source data of $t_{50\%}$ expressed in minutes at varying GC content percentages, used for the scatter plot in figure 4B (top).

	$t_{50\%}$ (min)			
GC content (%)	*Spy*dCas9	*Sp*RYdCas9	*Lb*dCas12a	imp*Lb*dCas12a
30.0	98	371	66	192
40.0	152	369	46	185
50.5	205	370	30	164
60.0	249	371	17	155
70.0	270	373	8.5	153

Source Data Table 7. Source data of the number of PAM sequences at varying GC content percentage, used for the scatter plot in Figure 4B (bottom).

	Number of PAM sequences			
GC content (%)	*Spy*dCas9	*Sp*RYdCas9	*Lb*dCas12a	imp*Lb*dCas12a
30.0	481,900.2	21,417,790	596,887	3,291,935
40.0	856,711.5	21,417,790	404,796.2	2,767,170
50.5	1,365,518	21,417,790	244,347	2,324,340
60.0	1,927,601	21,417,790	137,073.8	2038970
70.0	2,586,332	21,417,790	64,375.98	1,887,440

Source Data Table 8. Source data of target-bound fractions at different number of tracks per cell, used for the scatter plot of Figure 5C.

	*Spy*dCas9		*Sp*RYdCas9		*Lb*dCas12a		imp*Lb*dCas12a	
Apparent copy number	Target-bound	St. dev.	Target-bound	St. dev.	Target-bound	St. dev.	Target-bound	St. dev.
7-50	15	4	5	2	35	4	29	5
51-100	13	4	3	1	30	2	26	3
101-150	10	2	3.6	1	28	4	20	3
151-200	3	2	3.2	2.5	25	3	18	3

1. Leenay,R.T., Maksimchuk,K.R., Slotkowski,R.A., Agrawal,R.N., Gomaa,A.A., Briner,A.E., Barrangou,R. and Beisel,C.L. (2016) Identifying and Visualizing Functional PAM Diversity across CRISPR-Cas Systems. *Mol. Cell*, **62**, 137–147.

2. Thompson,M.G., Sedaghatian,N., Barajas,J.F., Wehrs,M., Bailey,C.B., Kaplan,N., Hillson,N.J., Mukhopadhyay,A. and Keasling,J.D. (2018) Isolation and characterization of novel mutations in the pSC101 origin that increase copy number. *Sci. Rep.*, **8**, 1590.

3. Jumper,J., Evans,R., Pritzel,A., Green,T., Figurnov,M., Ronneberger,O., Tunyasuvunakool,K., Bates,R., Žídek,A., Potapenko,A., *et al.* (2021) Highly accurate protein structure prediction with AlphaFold. *Nat. 2021 5967873*, **596**, 583–589.

4. Mirdita,M., Schütze,K., Moriwaki,Y., Heo,L., Ovchinnikov,S. and Steinegger,M. (2022) ColabFold: making protein folding accessible to all. *Nat. Methods 2022 196*, **19**, 679–682.

5. Fleming,P.J. and Fleming,K.G. (2018) HullRad: Fast Calculations of Folded and Disordered Protein and Nucleic Acid Hydrodynamic Properties. *Biophys. J.*, **114**, 856–869.

6. Globyte,V., Lee,S.H., Bae,T., Kim,J.-S. and Joo,C. (2019) CRISPR/Cas9 searches for a protospacer adjacent motif by lateral diffusion. *EMBO J.*, **38**, e99466.

7. Yang,M., Sun,R., Deng,P., Yang,Y., Wang,W., Liu,J.J.G. and Chen,C. (2021) Nonspecific interactions between SpCas9 and dsDNA sites located downstream of the PAM mediate facilitated diffusion to accelerate target search. *Chem. Sci.*, **12**, 12776–12784.

8. Bustin,S. (2015) Molecular Biology of the Cell, Sixth Edition; ISBN: 9780815344643; and Molecular Biology of the Cell, Sixth Edition, The Problems Book; ISBN 9780815344537. *Int. J. Mol. Sci.*, **16**, 28123.

9. Jeon,Y., Choi,Y.H., Jang,Y., Yu,J., Goo,J., Lee,G., Jeong,Y.K., Lee,S.H., Kim,I.S., Kim,J.S., *et al.* (2018) Direct observation of DNA target searching and cleavage by CRISPR-Cas12a. *Nat. Commun. 2018 91*, **9**, 1–11.
